# Doctors Believe Extermination of Malaria Possible

**Published:** 1916-01

**Authors:** 


### Doctors Believe Extermination of Malaria Possible.

Hopes for the extermination of malaria were expressed at the convention of the Southern Medical Association at Dallas on November 10th. No physician ventured to predict when it would be exterminated but several said emphatically it could be done.

Discussion of means of eradication centered mostly in methods of curing malarial patients completely and speedily without the necessity of long treatment, thereby preventing them from becoming carriers of the disease. A method of treatment which, although discarded years ago, has but recently come into much use, namely, the injection of quinine in solution directly into the veins, was advocated emphatically by several physicians as a long step toward eradication.

Dr. E. T. Wright, of Monroe, La., told of more than two hundred experiments with this treatment among employes of the Iron Mountain Railroad system, which has one of its principal hospitals at Monroe.

This treatment, he said, had shortened the average stay in the hospital when ill with malaria by two days. It more nearly sterilized the blood from infection than any other method, he said.

Dr. R. H. von Ezdorf, of New Orleans, of the United States Public Health Service, said he doubted the absolute results which seemed apparent from the railroad men's experience, and that the Monroe record was unusual.

Dr. Louis Le Roy, of Memphis, said experiments in that city, in part, bore out the good results obtained in Monroe. He told of experiments which he has just commenced to use a bichloride solution for direct injection into the blood.

In chronic cases of one selected type, he said, this remedy was producing initial good results.

---

Within the last twenty-five years the average length of man's life has been increased ten years, according to Dr. Victor C. Vaughn, Dean of the Medical College of the University of Michigan, who spoke before the Central States Conference on Problems of Social Hygiene. Dr. Vaughn said it is possible that humanity, if the present advance in medical learning is continued, will see the average length of life of the next generation increased fifteen years.

